# Glioblastoma Arising in an Immature Teratoma: A Rare Somatic Malignancy in a Germ Cell Tumor

**DOI:** 10.7759/cureus.109413

**Published:** 2026-05-22

**Authors:** Sagarika Pahwa, Sunipa Ghosh Pradhan, Soumendra Mishra, Sudipta Roy, Sandipan Chowdhuri

**Affiliations:** 1 Pathology, Saroj Gupta Cancer Centre and Research Institute, Kolkata, IND; 2 Surgical Oncology, Saroj Gupta Cancer Centre and Research Institute, Kolkata, IND

**Keywords:** germ cell tumour (gct), glioblastoma, high-grade immature ovarian teratoma, rare cancers of female genital tract, somatic malignancy

## Abstract

Mature cystic teratomas constitute the most common ovarian neoplasm occurring in childhood. All the components present in mature cystic teratoma are, by definition, mature. Immature teratomas, on the other hand, are malignant, and they constitute a mixture of embryonal and adult tissues derived from all three germ layers. Commonly, the main component is neuroepithelium. The treatment of immature teratoma is surgery and multidrug chemotherapy for grade 2 and 3 tumours. Sometimes only mature components persist in metastatic sites, which may continue to grow (growing teratoma syndrome) following chemotherapy. Emergence of benign or malignant neoplasm with somatic-type features is an uncommon event in mature cystic teratomas. The most common malignant change is squamous cell carcinoma, followed by carcinoid tumour and adenocarcinoma. Other types include melanomas, sarcomas of various types, carcinosarcomas, glioblastoma, etc. Glioblastomas are among extremely rare somatic malignancies arising from mature or immature teratomas. Their etiology is not well understood, and it is still a matter of research whether they share the same genetic alteration as the tumours occurring in the central nervous system. We present a rare case of glioblastoma arising in an immature teratoma in a young female in her early 20s.

## Introduction

Germinal epithelium covers the surface of the baby’s ovary. Germ cell tumors are derived from rudimentary germ cells of the embryonic ovary. A total of 20% to 30% of all ovarian malignancies are of germinal epithelial origin. The majority of them (95%) are benign and represented by mature cystic teratoma, while only 5% are malignant [1]. Mature cystic teratomas constitute the most common type of germ cell tumor, accounting for 60% of all ovarian neoplasms occurring in women less than 20 years [2]. They comprise mature tissues from all three germ cell layers (ectoderm, endoderm, and mesoderm), including cartilage, bowel, thyroid, skin, hair, teeth, and neuroepithelium. While mature cystic teratomas are benign, somatic-type malignancy occurs in up to 2% of these tumors, 80% of which are squamous cell carcinoma [3]. The ectoderm, endoderm, and mesoderm are the three germ cell layers that make up an immature teratoma, a germinal malignant tumour that is histologically identified by immature tissue, most commonly neuroepithelial tissue [4]. Either an immature teratoma or a teratoma with malignant neuroectodermal transformation may have immature neuroectodermal tissue in the ovary. Although their biological similarity is unknown, some lesions may mimic tumours of the central nervous system (CNS) [5]. Herein, we describe the case of a 24-year-old female who developed glioblastoma from an immature teratoma with widespread metastatic disease in the abdomen.

## Case presentation

A 24-year-old married female was getting treatment for primary infertility. Ultrasonography of the abdomen and pelvis revealed a mass in the right ovary, which was radiologically diagnosed as a dermoid cyst. She subsequently underwent laparoscopic ovarian cystectomy for the mass on 19/03/2025, which was diagnosed as an immature teratoma on histopathological examination at an outside center. The blocks were internally reviewed. 

**Figure 1:**
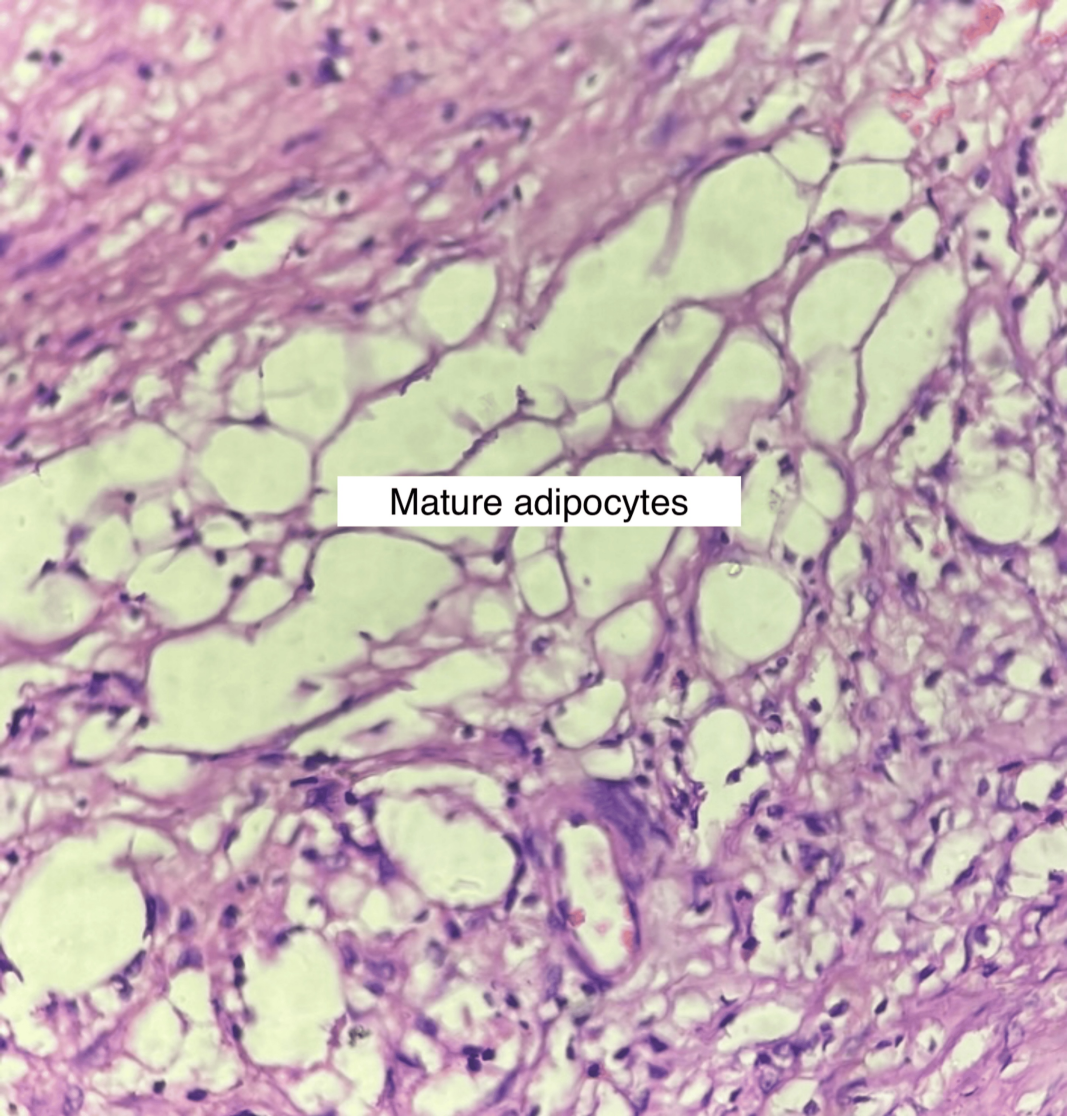
High-power image (40x): Haematoxylin and eosin-stained section showing mature adipose tissue.

**Figure 2:**
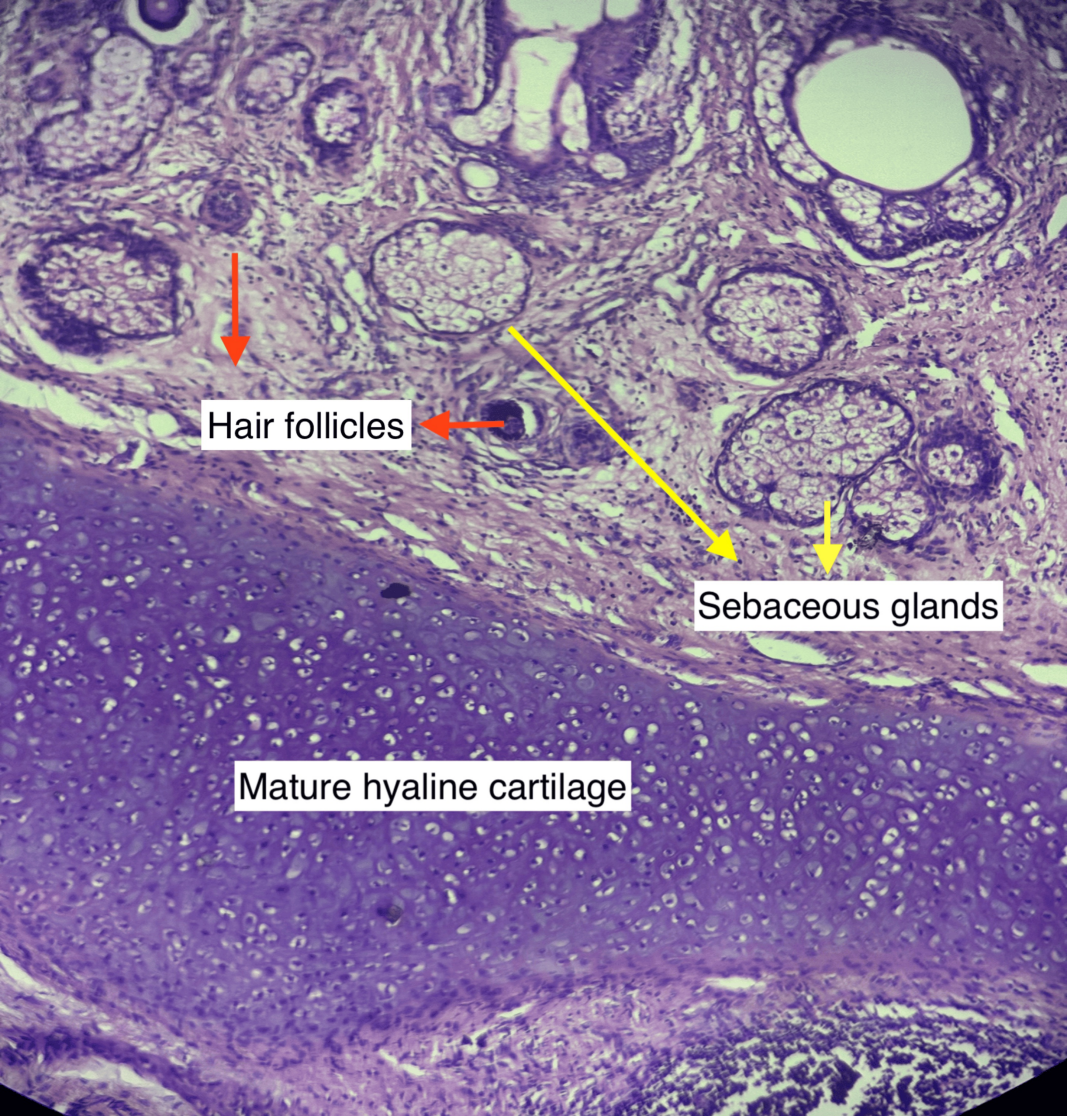
High-power image (40x): Haematoxylin and eosin-stained section showing mature cartilage and adnexal structures like hair follicles and sebaceous glands.

**Figure 3:**
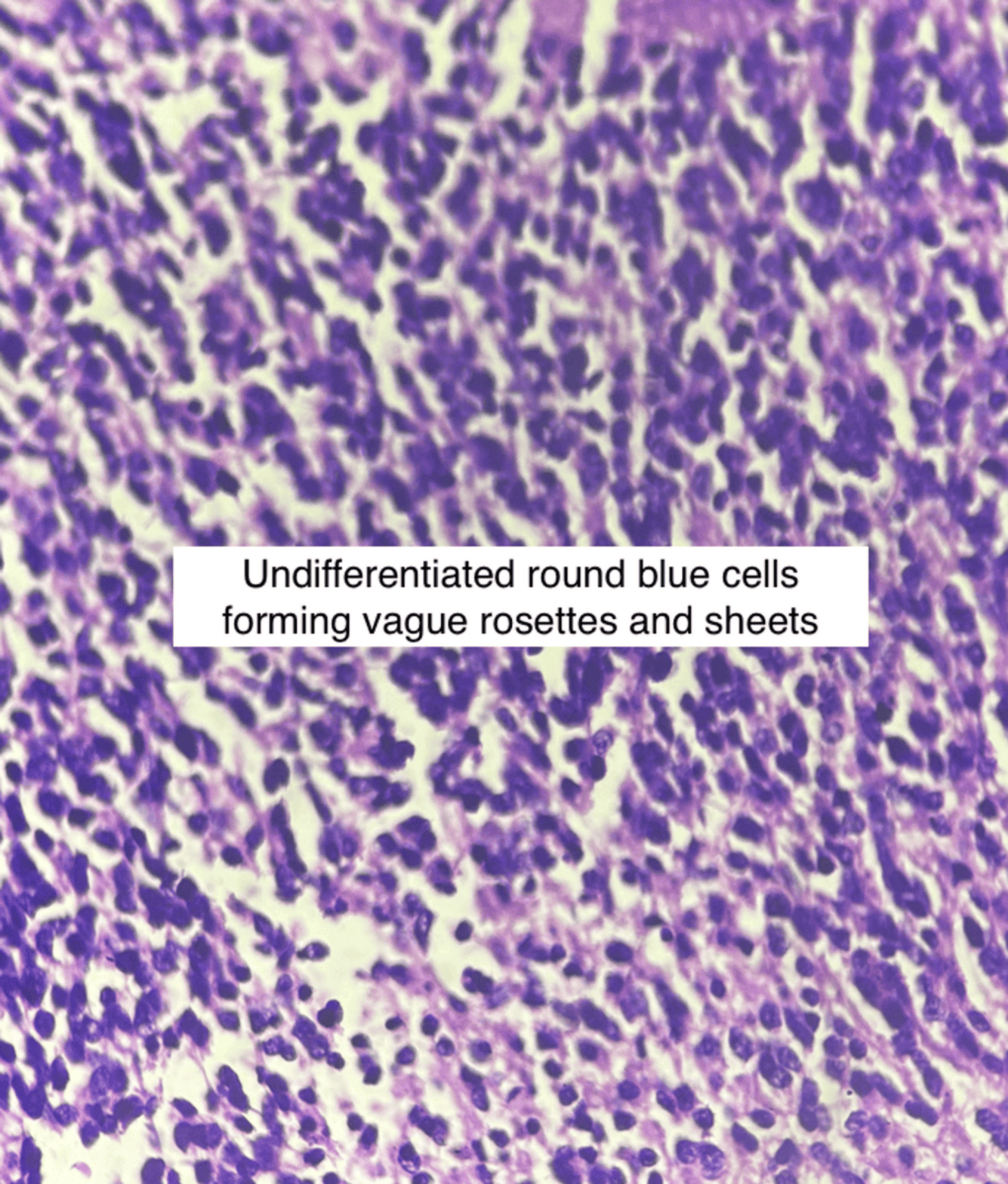
High-power image (40x): Haematoxylin and eosin-stained section showing sheets and rosettes of cells exhibiting near nil cytoplasm and darkly stained hyperchromatic nuclei, resembling immature neuroepithelial element.

Sections from the ovarian cystectomy specimen revealed myxoid, cartilaginous, lipomatous, glandular, and squamoid areas with adnexal structures. Admixed with these were areas of immature neuroepithelial elements having darkly stained hyperchromatic nuclei. They showed tubule and rosette formation. Mitoses were brisk. These neuroepithelial elements occupied an area of more than 3 high-power fields. Based upon these histomorphological features, the diagnosis of immature teratoma, grade 3, was reconfirmed (Figures 1-3).

Following this, she was given an adjuvant chemotherapy with bleomycin, etoposide, and cisplatin (BEP), followed by which she was planned for a cytoreductive surgery. Pre-adjuvant chemotherapy (14/05/2025) and post-adjuvant chemotherapy (21/07/2025) scans revealed disease progression with extensive peritoneal and nodal dissemination. A radiological scan of the head performed on 19/10/2025 did not reveal any significant intracranial abnormality.

The cytoreductive surgery was done at our institute (Saroj Gupta Cancer Centre and Research Institute) on 23/12/25, which included an en bloc resection of the uterus with both parametria and right ovarian mass, pouch of Douglas (POD) disease, pelvic peritoneum with rectosigmoid resection and anastomosis, gastric arcade sparing omentectomy, left salpingectomy, excision of peritoneal deposit over bladder, and loop ileostomy.

**Figure 4:**
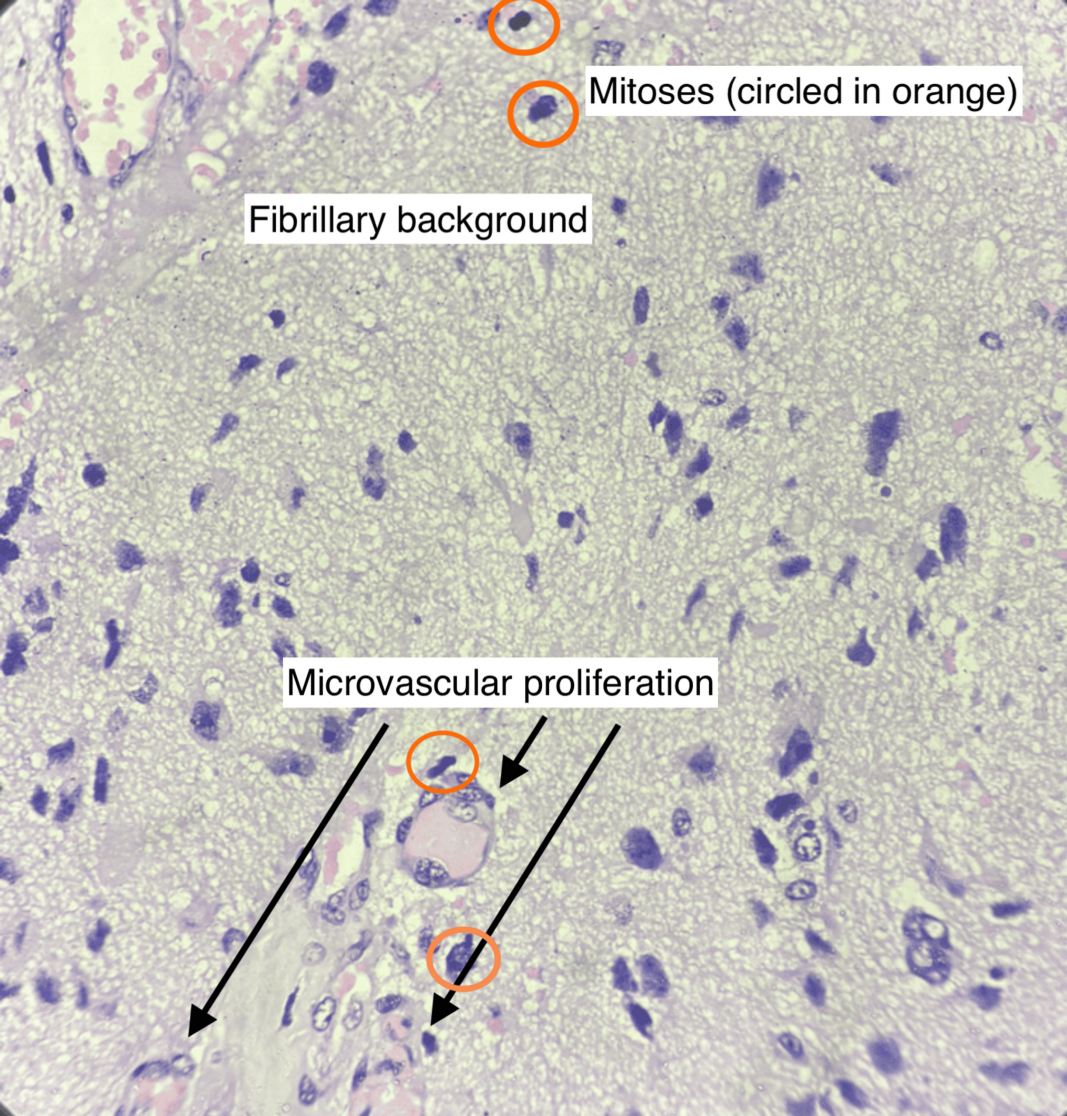
High-power image (40x): Haematoxylin and eosin-stained section showing pleomorphic tumour cells with hyperchromatic nuclei resembling astrocytes, lying against a fibrillary background. Foci of microvascular proliferation are also seen.

**Figure 5:**
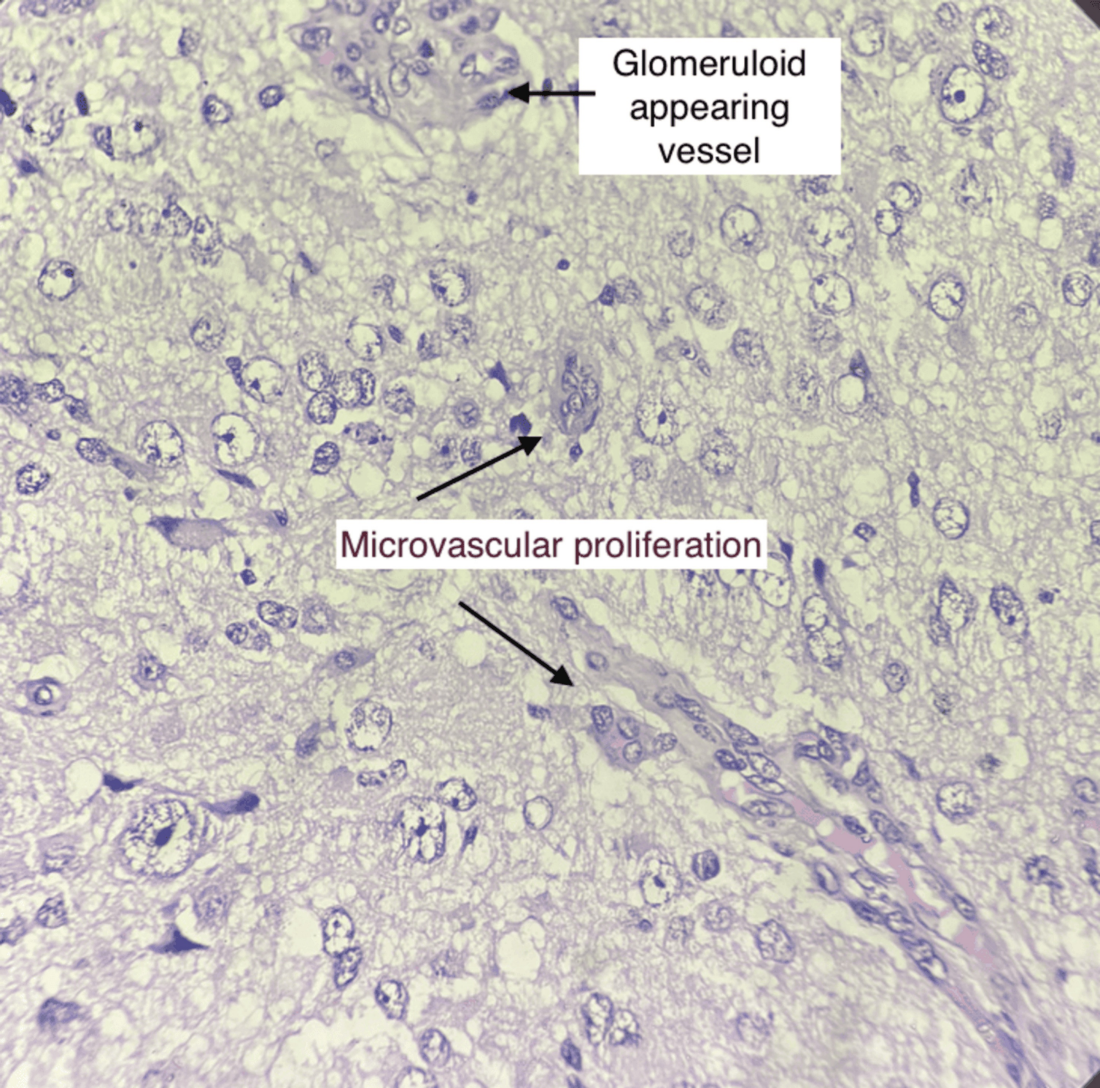
High-power image (40x): Haematoxylin and eosin-stained section showing pleomorphic tumour cells exhibiting epithelioid morphology with vesicular nuclei, prominent nucleoli, and mitotic activity along with foci of microvascular proliferation against a fibrillary background.

**Figure 6:**
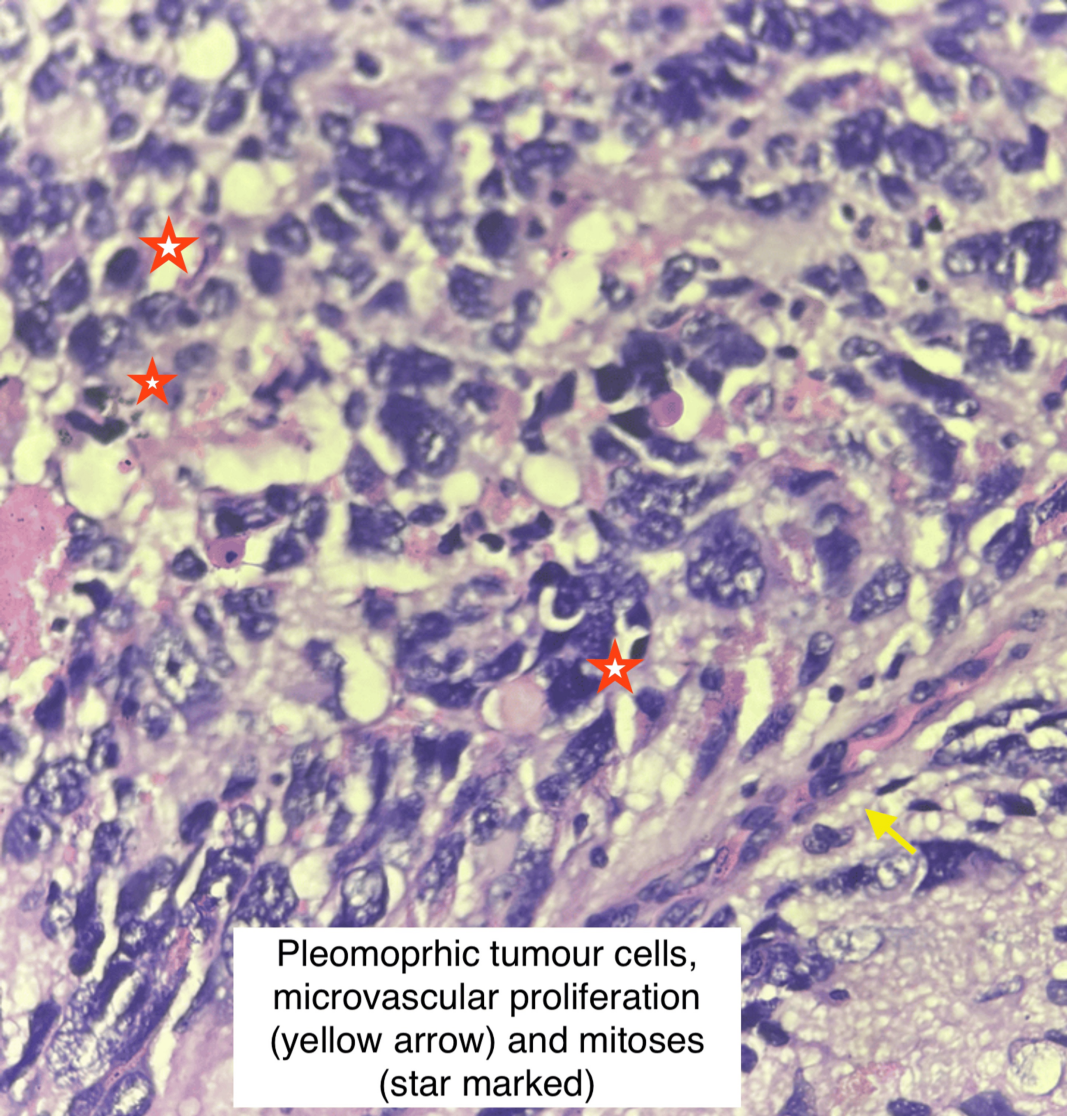
High-power image (40x): Haematoxylin and eosin-stained section showing sheets of darkly stained hyperchromatic pleomorphic undifferentiated looking tumour cells, reminiscent of primitive neuronal component.

**Figure 7:**
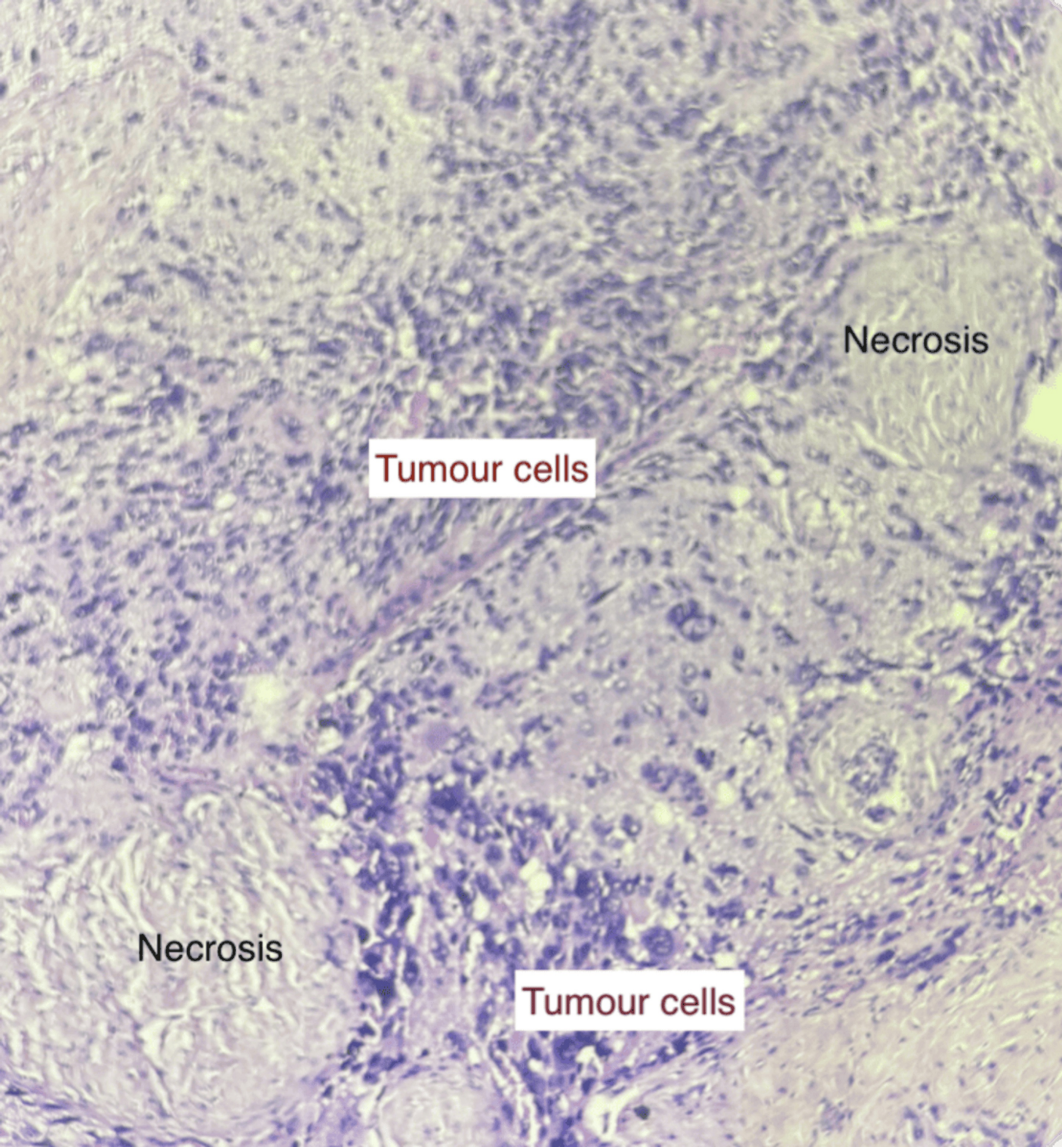
Low-power image (10x): Haematoxylin and eosin-stained section showing areas of necrosis admixed with tumour.

Multiple sections examined from the right adnexal mass showed a near complete replacement of the ovarian tissue by a hypercellular neoplasm composed of pleomorphic atypical cells lying against a fibrillary background. These cells had variable morphology ranging from primitive neuronal, gemistocytic, epithelioid, oligodendroglial-like, and astrocytic. Microvascular proliferation was extensive. Areas of necrosis were present along with atypical mitoses. Residual neuroepithelial elements were also identified. Omental lymph node, POD, rectal and bladder deposits, along with pericolic and perivesical lymph nodes, were involved by deposits of this neoplasm (Figures 4-7).

**Figure 8:**
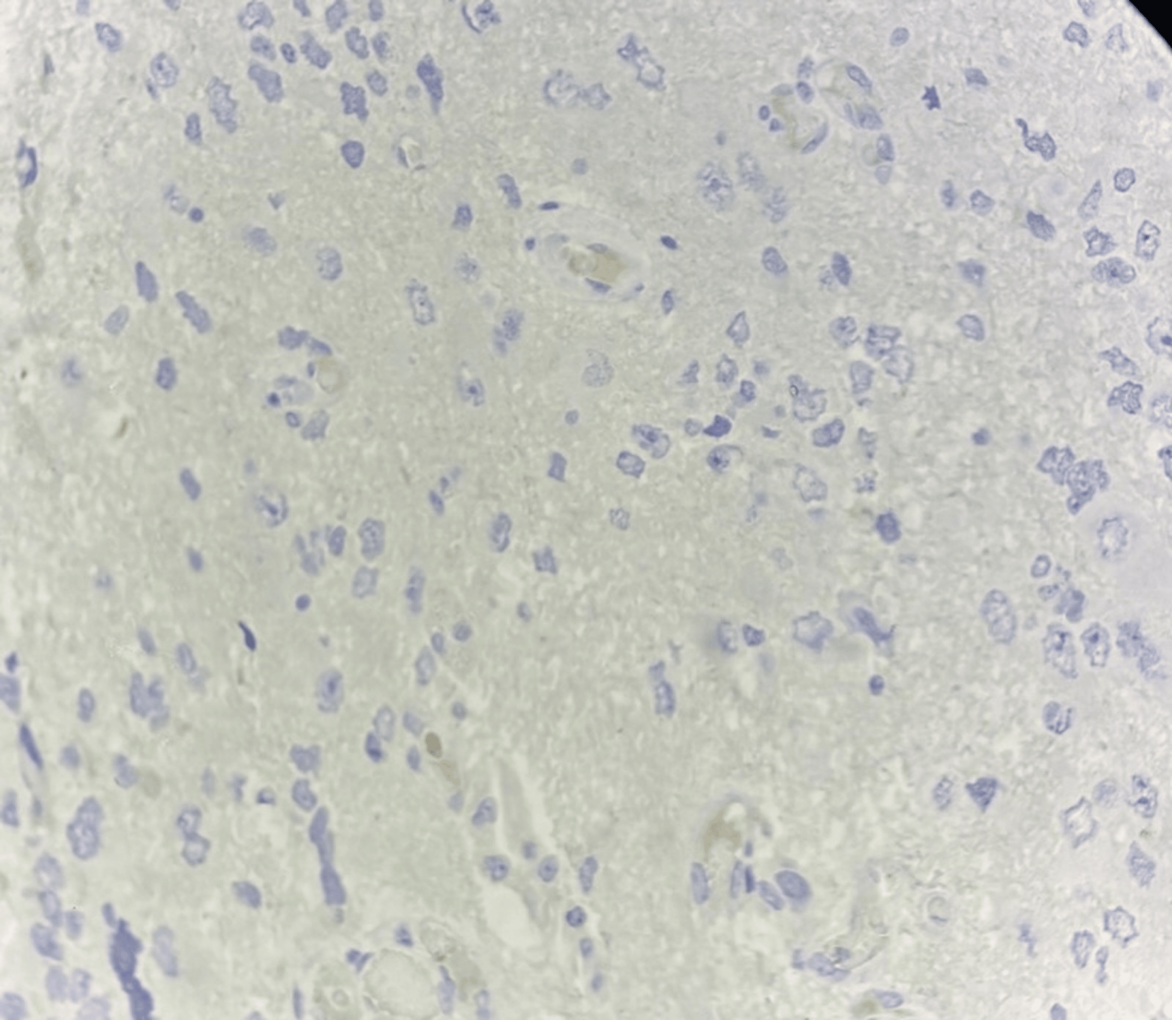
High-power image (40x): Immunohistochemistry for IDH1 (R132H) - negative. A next-generation sequencing (NGS) glioma panel done on the section was positive for a clinically relevant pathogenic mutation in the TP53 gene, whereas a reverse transcriptase polymerase chain reaction (RT-PCR) for O6-methylguanine-DNA methyltransferase (MGMT) gene did not detect any promoter methylation. IDH1: isocitrate dehydrogenase 1

Immunohistochemistry done on the sections showed tumour cells to be positive for glial fibrillary acidic protein (GFAP) (strong and diffuse), neuron-specific enolase (strong and diffuse), S100 (strong and patchy), and p53 showed strong and diffuse nuclear staining. The tumour cells were negative for pancytokeratin, p40, synaptophysin, glypican 3, and CD30. The Ki-67 index was approximately 40%. Immunohistochemistry for isocitrate dehydrogenase 1 (IDH1 (R132H)) was negative (Figure 8).

A next-generation sequencing (NGS) glioma panel done on the section was positive for a clinically relevant pathogenic mutation in the TP53 gene, whereas reverse transcriptase polymerase chain reaction (RT-PCR) for O6-methylguanine-DNA methyltransferase (MGMT) gene did not detect any promoter methylation. The slides were reviewed by experts, and a diagnosis of high-grade glioma consistent with glioblastoma was made, arising in a known case of immature teratoma.

## Discussion

Only a small number of glial and neuronal tumours linked to or subsequent to numerous embryonic teratomas have been reported. Neuroectodermal tumours in the CNS are similar to these uncommon tumours [6,7]. It is extremely uncommon for glioblastoma multiforme to develop inside a teratoma and for intra-abdominal glioblastoma multiforme to be accompanied by gliomatosis peritonei. The rarity of glioblastoma developing in a mature ovarian teratoma calls for research into any underlying genetic susceptibility, as was noted by den Boon et al. [8]. It's unclear what the molecular signature of secondary glioblastoma that develops from an ovarian mature cystic teratoma looks like. It is unclear if the absence of an IDH mutation is a feature shared by all ovarian glioblastomas, even though the morphology of these tumours is similar to that of CNS tumours of the IDH-wild-type [9]. The typical IDH1 R132H mutations observed in CNS diffuse astrocytomas are not present in primary ovarian astrocytomas, despite their morphologic resemblance. In contrast to the CNS, where this is usually not possible, ovarian glial and neuronal tumours can be surgically extensively debulked and frequently totally removed [10]. On the other hand, it was observed that p53 expression is not unusual in immature teratoma, and diffuse p53 immunopositivity is associated with recurrence or the presence of malignant elements in approximately 50% of cases of immature teratoma [10]. Despite their morphologic and immunophenotypic similarities, primary glial and neuronal tumours of the ovary and peritoneum differ molecularly from their counterparts in the CNS, according to a study by Liang et al. [3,11]. Cytoreductive surgery is part of the first-line treatment for ovarian glioblastoma, unlike treatment for brain glioblastoma. For low-stage tumours, surgery alone may potentially be curative [12]. Some patients were able to achieve disease-free survival after a course of adjuvant chemotherapy with BEP [13]. In our case, these chemotherapeutic drugs were used as adjuvant therapy, despite which extensive residual disease remained because the patient had already progressed to a higher stage. Early-stage cases may be treated with cytoreductive surgery alone; advanced-stage cancers demonstrate aggressive behaviour and require additional therapy. The optimal adjuvant treatment regimen remains unknown, and the response to treatment may differ from glioblastoma of the brain. Despite the adjuvant therapy course, the overall survival of advanced-stage ovarian glioblastoma remains poor [13].

## Conclusions

Glioblastoma is among the exceedingly rare somatic malignancies encountered in mature or immature teratomas. Despite having morphological similarity with CNS glioblastoma, the similarity in their genetic makeup has not been established, thus differing in their treatment modalities.
